# Exploring Cultural Competence amongst OT Students

**DOI:** 10.1155/2017/2179781

**Published:** 2017-08-09

**Authors:** Pragashnie Govender, December M. Mpanza, Tarryn Carey, Kwenzile Jiyane, Bicolé Andrews, Sam Mashele

**Affiliations:** Discipline of Occupational Therapy, School of Health Sciences, University of KwaZulu-Natal, Westville Campus, Westville, South Africa

## Abstract

Occupational therapy relies primarily on communication between the therapist and client for effective intervention. Adequate communication may be influenced by language and cultural differences between the therapist and client. Cultural competence in relation to language and culture is thus a vital part in practice. Limited research exists on cultural competence in occupational therapy students. This study thus aimed to explore the cultural competence of final year students and their perceptions of their own cultural competence, with respect to language and culture in their practice as students. An explorative qualitative study design was utilised with a nonprobability purposeful sample of 21 final year undergraduate students at a tertiary institute in South Africa. Three focus groups were conducted, comprising between 6 and 8 students in each group. Thematic analysis using inductive reasoning was undertaken in order to analyse the students' experiences and understanding of cultural competence. Findings of the study suggest that cultural competence, in relation to language and culture, influences the occupational therapy intervention process. It was shown to both positively and negatively influence intervention through supporting or hindering rapport building, client centeredness, and effective intervention.

## 1. Introduction

The concept of cultural competence, which in this study relates to the understanding of the influence of different languages and cultures, forms an essential component in the practice of any health care profession. Clear communication is necessary, between the clinician and client, for exchange of vital information for effective intervention [1]. Occupational therapy is one such profession which relies primarily on the interchange of communication between the therapist and client. Clients are viewed as unique beings, where their difficulties are as a result of a combination of factors and where the management of the client may rely on an understanding of the client's culture and family, including continued communication and/or support with the client and family [2]. Communication difficulties resulting from language and cultural differences between the therapist and client can thus have a significant impact on cultural competence and the occupational therapy intervention process. This is of concern in the South African context, given the country's multicultural population and eleven official languages. According to Penn [3], more than 80% of interactions between physicians and patients in South Africa take place across linguistic and cultural differences and therefore directly influence the patient-therapist relationship and quality of care. It is thus imperative that health practitioners and occupational therapists in general bridge these gaps in language and cultural differences in practice to gain a sense of cultural competence. To the authors' knowledge there has been only one other study conducted on cultural competence amongst student occupational therapists within South Africa [4] and one other study on cultural competence on professional South African occupational therapists [5]; thus limited evidence exists on how therapists in South Africa view language and culture as influencing practice and how students perceive and negotiate cultural competence in practice.

## 2. Literature Review

Occupational therapy is a diverse profession which relies on a functional relationship with the client whilst examining the physiological, psychological, and environmental components that are unique to that person [1]. Intervention is thus more effective when the therapist can communicate with the patient about treatment [1]. Effective communication results when all participants in the communication process understand the content of what is being communicated [6]. A therapist speaking a different language to their client would thus have difficulty in fully grasping the client's cultural context without fully understanding the client's language first. Language is also a primary purveyor of culture. A study conducted by Levin [7] found that there was a link between language and culture. If language becomes a barrier between the practitioner and client, then the difference in their cultures could become a barrier as well. In situations such as these, cultural sensitivity needs to be applied. Moore [8] emphasised that to be culturally sensitive, one needs to be willing to consider the cultural background of the client and have at least the basic knowledge of the client's culture or beliefs within the society they practice. The Occupational Therapy Association of South Africa (OTASA) Code of Ethics and Professional Conduct [9] indicates that “occupational therapy services shall not allow prejudice or discrimination towards a patient/client based on race, culture, language, age, sexual orientation, disability, socioeconomic status.” Ulrey and Amason [10] suggest that health care providers may not be aware of the client's cultural values, beliefs, and attitudes and learn as they experience it within the field of practice to become culturally competent.

**Figure 1:**
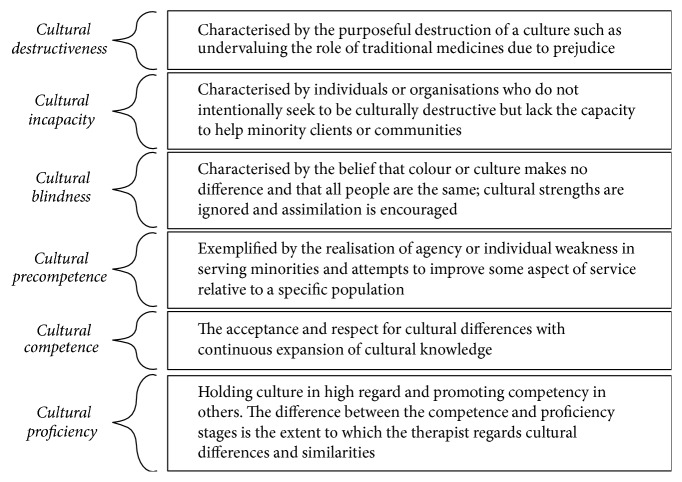
Cultural competence continuum [12, 13].

Cultural competence is defined as “the process by which individuals and systems respond respectfully and effectively to people of all cultures, languages, classes, races and ethnic backgrounds in a way that recognizes, affirms, and values the worth of the individual and protects and preserves the dignity of each.” [11]. It is thus imperative for students to be sufficiently exposed to different cultures and languages to build cultural competence which can be a lifelong learning process. Cultural competence, according to Cross [12], is described in six stages through which an individual develops along a continuum from cultural destructiveness to cultural proficiency. The stages are further defined as per Wittman and Velde's [13] definition of each stage (Figure 1).

Therapists in any of the first three stages, that is, destructiveness, incapacity, or blindness, may be considered to be culturally incompetent [13]. Evidence indicates that lack of cultural competence in the therapist can impact on therapeutic outcomes, treatment implementation, compliance, and follow-up [14]. Cheung et al. [15] conducted a study assessing perceptions of cultural competence amongst 51 occupational therapy students in England, in which they found a majority of students having reported limited awareness of and exposure to other cultures and insufficient training in cultural competence.

Cultural competence requires the health care practitioner to understand his/her own cultural values before understanding other cultures, their communication, behaviour, and the impact of their culture on the individual's health care beliefs. Language and cultural barriers are a common concern in clinical practice both in South Africa and internationally. With communication difficulties between the patient and the health care provider, there is often the use of interpreters either trained or untrained which could result in treatment being effective or ineffective.

A study conducted in the western United States explored the perspectives of 495 primary care physicians and their use of various methods to bridge language and cultural barriers between themselves and their clients [16]. In this study, authors found that in a place of cultural and ethnic diversity where physicians had access to the services of trained interpreters, a significantly higher quality of patient-physician communication and care occurred. Health care providers are exposed to diverse cultures and experience challenges to providing culturally and linguistically appropriate care to result in cultural competence. Occupational therapy as a profession thus needs to focus on providing culturally and linguistically appropriate care in order to provide holistic and culturally competent intervention.

## 3. Material and Methods

An explorative qualitative study design was utilised to explore occupational therapy undergraduate students' perceptions of cultural competence in relation to language and culture and the influence on their practice in the province of KwaZulu-Natal in South Africa. Nonprobability purposive sampling was used to select the study population of 24 final year undergraduate occupational therapy students from the school of health sciences at a tertiary institution in the province. From this sample, 21 participants agreed to participation in the study. The target population was comprised of a diverse group of students that speak different home languages and who hail from variable cultural backgrounds. Occupational therapy final year students were selected as they would have had at least three years of exposure to various service learning sites as part of their occupational therapy undergraduate training at the time. These service learning sites that the undergraduate students are exposed to during training are multicultural settings comprising hospitals, clinics, community sites, rehabilitation facilities, geriatric homes, and schools, serving a culturally and linguistically diverse population.

Three focus groups comprising a maximum of eight students were conducted spanning approximately one hour. Participants were divided into engineered heterogeneous focus groups to ensure cultural diversity. Questions posed to the groups included the following:

- The experience of students working with clients of a similar and divergent language and culture compared to their own.
- The student's opinions on the influence they believed language and culture have on occupational therapy practice/intervention.
- The opinion of students on the positive and negative aspects that language and culture have on their OT practice and how they dealt with this in practice.
- Student's understanding of the term cultural competence.
- Student's opinions of their preparedness to deal with clients of varied cultures and language backgrounds.
- Student's learnings of language and cultural influences in their practice and how they have negotiated this in OT practice.

Students responded to these questions and shared their fieldwork experiences of the past three years. Furthermore, probes were used to elicit deep reflection of their personal experiences rather than actual observations during fieldwork.

Audio voice recordings of each focus group, with participant consent, occurred and data were transcribed verbatim by authors. Transcriptions of the focus groups were proof-read by all authors prior to being analysed, to ensure investigator triangulation of results. Qualitative data collected from the focus groups were then thematically analysed using inductive reasoning. The process of thematic analysis involved the use of four levels of coding, including initial codes, creation of subcategories, categories, and themes. Trustworthiness was achieved through examination and comparison of data collected to existing bodies of knowledge on the topic (credibility). Transferability was achieved through the authors' documentation and justification of the methodological approach and the critical processes and procedures that helped them to construct and create meaning associated with the study. Dependability was achieved through regular peer briefings which ensure analytical triangulation. These strategies allowed for validation and congruency of research findings by all team members. To ensure confirmability steps were taken by the authors to ensure that the study findings are representative of the experiences of the participants' rather than being influenced by the researchers [17]. Four of the authors were final year occupational therapy students at the time of the study. Therefore, steps were taken to reduce bias; these included bracketing through reflexive journaling prior to and during data collection and triangulation during the data analysis process.

Ethical clearance was obtained from the University of KwaZulu-Natal Research Ethics Committee (Ethical Approval number SHSEC 007/16). Additionally, consent to access students in this study was obtained via gatekeeper permission from the registrar of the institution. Prior to consent from participants, an information document stating participant's rights, ethics, aims, and procedures of the study was forwarded to the participants. Those that volunteered to participate signed informed consent and were assured of confidentiality and no physical or emotional risk to them and that that they could withdraw from the study at any time.

## 4. Findings

**Figure 2:**
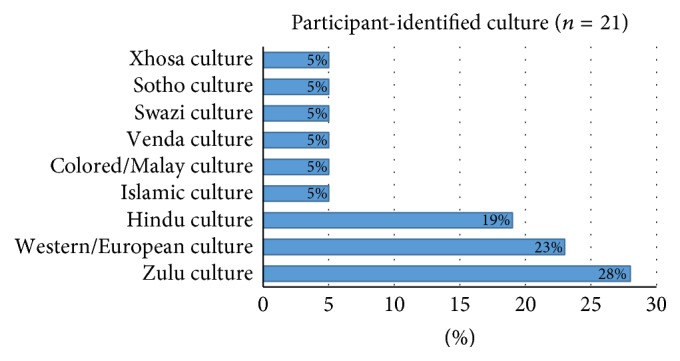
Participants identified culture (*n* = 21).

**Figure 3:**
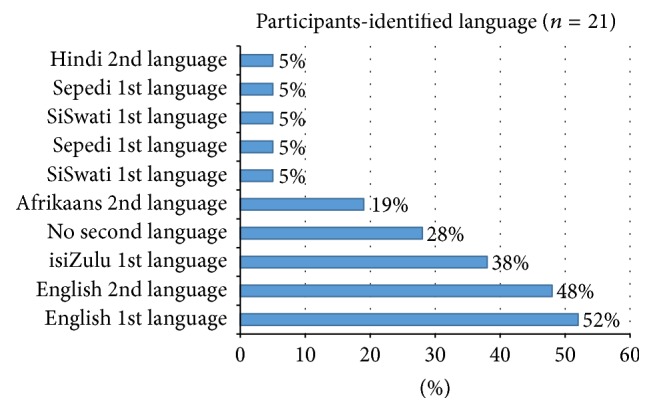
Participants identified language (*n* = 21).

*Demographic Profile of OT Students*. Of the 21 students that participated in the study, 19 (90%) were female and two (10%) were male. The median age of the participants was 20 years. In terms of home language, 11 (52%) participants were English-speaking, eight (38%) spoke isiZulu, one (5%) spoke SiSwati, and one (5%) was Sepedi-speaking. Of the participants, 10 (48%) spoke English as a second language, 4 (19%) spoke Afrikaans as a second language, one (5%) spoke Hindi as a second language, and six (28%) identified themselves as having no second language. Identified cultures and language of participants are highlighted in Figures 2 and 3, respectively.

**Figure 4:**
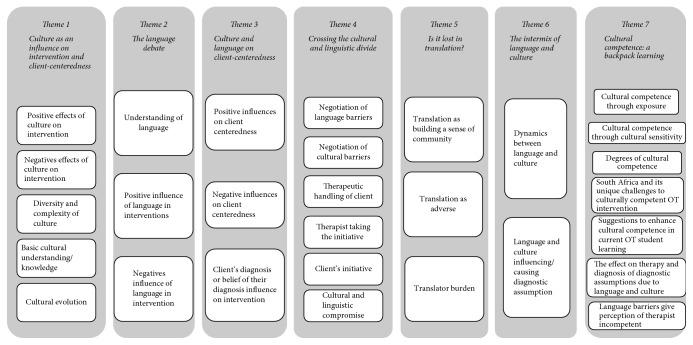
Themes and subthemes.

Seven themes emerged, of which many were common to the content of the reflexive journals of student authors. These included “culture as an influence on intervention,” “culture and language on client centeredness,” and “is it lost in translation.” Refer to an overview of themes presented in Figure 4 and described in detail below.

**Theme 1 (culture as an influence on intervention).**

Participants described culture as* “a mixture of beliefs,”* one's traditions, values, and interests.* It is “how we explain things, how we make sense of the world and everything…around us” *(Thami). This description was similar to the views expressed in the reflexive journal of four student authors which were documented prior to commencing data collection. As students, participants indicated that they had been exposed to diverse cultures both in the lectures and during service learning which had broadened their cultural knowledge and understanding:* “When I got a Zulu patient, I got to learn more about my culture, about how they…do their practices instead of how I do at home” *(Maggie). Sadie expressed that* “Even though they are the same colour as me does not mean they are the same culture.” *Ashrena added that* “It gives you the opportunity to go back and learn about them and that's just increasing your knowledge into different cultures.”*

Culture was further identified by participants to influence intervention both positively and negatively.* “There is some sort of emotional attachment…with people of the same culture”* (Thami) and* “They sort of take advantage of the fact that they are similar”* (Ashrena). Four student authors shared the same view; they identified culture to have a strong influence in the OT intervention.

Participants indicated that they acknowledge that culture itself is evolving. It is* “very dynamic, it changes and it differs”* (Fatima) from individual to individual and as they have grown and evolved, so has their perceptions of culture:* “…culture is changing as it goes…changing all the time” *(Sadie).

**Theme 2 (the language debate).**

Participants understood language as the way in which they communicate and understand; it is the words and gestures used to get a message across. As Thami indicated* “…that one has a problem*…*I was like my goodness, the kid barely speaks…and then somebody one day was just like, oh ya, she's not from here." *

Participants, furthermore, found themselves frustrated by the linguistic barriers experienced.* “It's frustrating, not just frustrating for you, it is also frustrating for the person because they want to understand what you telling them…So it's a mutual frustration”* (Fatima).

Contrastingly, language for some participants had a positive impact on intervention; it assisted in building rapport with clients and had even resulted in participants learning a new language in order to connect to their clients.* “…It almost brought us together that all of us were talking different languages because we were all like trying to find a base that we could work from” *(Ashley). Sadie added* “working with a different language is it forces you to try and learn that language.”*

**Theme 3 (culture and language on client centeredness).**

Some participants viewed cultural and linguistic understanding within the South African context as aiding client-therapist rapport and understanding of their client's context within practice for more client directed therapy:* “Activities that we choose at the end of the day some of them have to be client centred and the only way you gonna know what your patient likes or doesn't like is if you actually communicate with them. So to make your therapy more client directed rather than therapist directed” *(Zandile).

However, majority of participants including the student authors viewed language and culture as detrimental to client centeredness when disregarded in intervention:* “I'm not familiar with games others (cultures) do. So that affects my plan of my treatment and it tends not to be client-centred but more of therapist-centred” *(Prince). Participant's further expressed that clients found comfort in familiarity which positively influenced client centeredness:* “The patient sort of gelled more with someone of the same culture as themselves… a familiarity thing” *(Thami). On the other hand, differences between the client's need and the therapists goals would result in the therapist's disregard of client centeredness:* “Because they (the client) are in need of something from you as a therapist and so they just accept whatever you may come with” *(Prince). The client's cultural and health beliefs were also perceived by participants as affecting client centeredness as one student reflects on an interaction with a client:* “…that man from Ubhumbulu sent a lightning bolt and I had a stroke so I need to go to Inyanga* (*Inyanga* is a traditional healer using cultural practices to speak to ancestors)* to cure” *(Thami). The participant was thus concerned that this cultural practice will affect interventions and acceptance of the therapeutic management offered.

*Theme 4 (crossing the cultural and linguistic divide)*. Participants made use of various strategies to negotiate language and cultural barriers which included translators, demonstration, effective handling of the client, learning written phrases, using digital applications for translation, researching language and culture, engaging with colleagues, and increasing intervention time to compensate for language barriers. Participants expressed the following:

A lot of it is done through body language um obviously if you get a translator that is amazing, on community I got another one of the students to write down the different instructions for me. (*Ashley*)

I've learned (about) culture from my classmates. (*Ashley*)

I'll go use the internet as a resource, books and other people of that culture so I just research about that. (*Sadie*)

A small percentage of participants stated that research alone was not enough to bridge the cultural and linguistic gap: As Sipho indicated,* “Like do some research on it…you always get told that and then you go to Google and find a book and there's never any information about it and how it impacts therapy.”*

Majority of participants, however, viewed themselves as personally responsible for taking the initiative to learn the culture and language and meet the client halfway:

It's up to me if I want to go deep or I want to be superficial. (*Sthabile*)

You bring yourself down to their level, there's no ‘my language' ‘my culture' all of that, that's the thing, I don't think it's up to the client, I don't think it's up to the OT, I think it's up to both of you to try get there cause if one of you is not willing then it's not gonna work. (*Ashley*)

*Theme 5 (is it lost in translation?)*. Participants and two student authors highlighted the influence of translators in therapy in their attempt to be culturally competent. Translators were mostly seen to have a negative impact when attempting to build a therapeutic rapport with clients as well as resulting in therapists' exclusion which negatively influences therapeutic use of self in therapy.

You losing the intervention you doing coz they (the translator) then end up almost taking over. (*Sipho*)

…the caregiver just always looks towards the translator and they actually never build that connection with you…(*Shanon*)

Sometimes as a therapist you happen to go out of the loop which a therapist should never be. (*Fatima*)

The burden placed on the translator, as viewed by the participants, was a further negative to translation:

…they say the long things…I'm not gonna remember everything, so I take the short cut and end up not giving the full message. (*Maggie*)

It is difficult especially when they have their own client to treat, and they are taking the time out of all the stuff that they need to do. (*Sipho*)

*Theme 6 (the intermix of language and culture)*. Throughout the focus group discussions, participants revealed that they view language and culture as synonymous with each other or as having a dynamic influence on each other:* “There is definitely a link between language and culture even though they are 2 separate things” *(Sadie).

Participants found that they could bridge the cultural gap with language:* “To communicate with patients in their language it helps you understand their context and once you understand their context you're able to go deeper into their culture”* (Thando).

*Theme 7 (cultural competence: a backpack of learning)*. Opinions of students varied on what it meant to be culturally competent, with majority of participants perceiving themselves as culturally incompetent, but with a willingness to engage in lifelong learning and growing towards the ideal of cultural competence:* “I don't know if you can ever be fully culturally competent but I feel like you can aspire towards cultural competence by using cultural sensitivity…it is something you should always work towards” *(Ashley). Penny indicated* “I don't even think that I am competence (competent) in all areas in my own culture.”*

However, participants still displayed cultural awareness, growth, and sensitivity due to exposure to and learning of different languages and cultures:* “I am definitely cultural sensitive*…*I don't just barge in with who I am and what I bring” *(Sadie). Ashrena added* “As you grow with the degree and learn about more culture and you become more emotionally attached to other cultures as well.” *

Majority of students felt that they had been provided with appropriate information and exposure to cultures and language during their training but some students including student authors still viewed this as insufficient:* “We get into the OT course and then we experience all these cultural forms and diversity, but it's not deep enough, we don't know deep enough” *(Sthabile). Phindile felt that this process is complex,* “…for the course teaching us about being competent, it's not that simple or easy.” *

Cultural competence in occupational therapy practice specific to the South African context was found to challenge students:* “I also think with the tests we have…they are like not adapted for everyone…they don't incorporate everyone there is in South Africa”* (Prince). Sarah added* “It has been tough working with such a diverse population.”*

## 5. Discussion

The findings of this study indicate that the student cohort who formed the study sample have been exposed to varied cultures through their undergraduate training. Cultural differences were found to negatively affect intervention, where, in some cases, clients refused intervention due to the cultural difference thus causing frustration for both the participants and their clients. These findings are aligned to other findings from a study by Mabuza et al. [18] on community placement areas of South African Universities, in which he demonstrated that, in some instances due to cultural or religious norms, treatment was often refused. Additionally, a study conducted by Levin [19] identified that cultural misinterpretation occurred, so did negative attitudes between the therapist and client, in some therapist-client encounters. However, culture was viewed by participants as having positive implications on intervention where it had improved rapport, intervention, and therapist's cultural knowledge, through exposure to various cultures. Consequently, this was influenced most often by the therapist's initiative in the interventions and in establishing rapport with clients that they encountered.

Language is considered to provide the means by which humans communicate, and communication plays a pivotal role in the health profession in developing trust and cooperation between the practitioner and client [20]. This is in keeping with findings of this study which highlighted that when trying to speak the client's language, participants noted positive changes in the client-therapist rapport building. It forced participants to learn a language to improve their intervention. Language was still, however, the greatest concern of the participants as this barrier negatively impacts intervention and their ability to be culturally competent. Participants found therapeutic opportunities were missed and the intervention provided was superficial or even incorrect. The findings are consistent with those of Ong et al. [21] and Schlemmer and Mash [22] who found that patient care and clinical outcomes suffered due to the language barrier. It is noteworthy to the authors to note the lack of distinction between language and culture in the study where students viewed both of these concepts as one entity or as one influencing the other. This corresponds with the literature that highlights that language is a primary purveyor of culture and the connection between language and culture is extensively rooted [20].

Occupational therapists view clients holistically, taking into account their linguistic and cultural context in order to create a client centred intervention. Majority of participants showed that when communicating effectively they were able to provide client centred intervention which was similarly found in a study conducted on occupational therapy students in the University of Free State in South Africa [4]. It is of a concern that some participants struggling with linguistic and cultural understanding were unable to provide the client centred therapy they felt their clients deserved. Furthermore, failure to consider a client's identified problems was noted by a few participants resulting in inaccurate history taking and misinterpretation of diagnosis which has also been well documented in the literature [23, 24]. This disregard for client centeredness, in most of the cases reported, would result in the acceptance of the student's cultural incompetence by the client due to client desperation and need for their help. It is thus important that practitioners find a way to cross the linguistic and cultural divide to meet the needs of the people with whom they work [7]. Whilst therapists try to bridge the cultural divide, they may consider that clients often “warm up” to therapists of similar culture which positively influences client centeredness as noted in this study. This could be essential where rapport is difficult to build. Furthermore, in bridging the gap between language and culture, cultural brokering which serves as a mediator is said to be valuable [25]. Responses from participants revealed the use of this technique as well as various other measures to bridge the gap of a cultural or linguistic divide, which included use of translators, use of research, and use of body language. Using translation was found to be useful in intervention for some, however, majority reported that it resulted in dilution of the information and therapist exclusion from the intervention process. Other studies [11, 26] also found that the use of translators affected rapport and accuracy of data being translated making it superficial, thus, impeding on assessment and intervention outcomes. The most notable finding was in the ability of students to take the initiative to learn the language of their clients for improved therapeutic outcomes. This is similar to a study by Tjale and De Villiers [27] that highlighted that self-empowerment in terms of culture and language was a factor that enables the health practitioner to provide effective intervention, as this encourages client's willingness to participate and influences positive rapport with clients.

Overall this cohort of students showed cultural sensitivity and understood cultures as diverse, forever evolving, complex, and specific to each individual. This alone showed a high level of precultural competence according to Cross [12] cultural competence model. Students however viewed themselves as generally culturally incompetent. Cheung et al. [15] study found that the majority of students reported having limited exposure to other cultures and insufficient training in cultural competence.

Two similar studies [4, 5], from a South African perceptive, also showed that students felt they did not have enough exposure to cultural issues in both university-based education and fieldwork. Students in these studies did not feel sufficiently equipped with respect to cultural competence as a result of the occupational therapy curricula at their institutions. These findings are in contrast to the findings of this study as participants suggested they had felt prepared through exposure and training at this particular institution. Wohl [28] suggested that health practitioners may not fully know all their client's cultural customs and attitudes; they become equipped through experience as they become exposed. These findings [28] support the participant's responses that cultural competence may not be fully obtained but enhanced through exposure to various cultures and that it is a continuous learning journey of collecting knowledge as they experience it, carrying this knowledge into all their practice, as expressed by one participant* “it's like a little backpack you carry with you everywhere”.*

## 6. Conclusion

In this study, the authors aimed to explore the perspectives of final year occupational therapy students on the issue of cultural competence. Whilst occupational therapy students are exposed to diverse languages and cultures in various fieldwork placements within their undergraduate training, students still viewed themselves as being culturally incompetent. Instead, students displayed cultural sensitivity, due to the belief that culture and its many subcultures are too diverse and evolving to ever reach an ideal state of cultural competence. Cultural incompetence related to language barriers and misunderstanding of the client's culture have been viewed as negatively influencing the intervention process specifically in rapport building, accurate assessments, and client centred activity choices. The negative influence of cultural incompetence could be exacerbated when attending to difficult clients. However, clients of similar culture to their therapists often build rapport easily; therefore, situations where therapists of similar culture act as mediators can be considered a step closer to client centeredness intervention. The authors found that participants use the term language and culture interchangeably indicating a strong perceived link between language and culture as influencing their ability to achieve cultural competence and client centred intervention. Participants thus viewed it as essential to take responsibility or initiative to deal with linguistic and cultural barriers to achieve cultural sensitivity and strive towards cultural competence. Negotiation of barriers was found to include body language, use of translators, research on the Internet, and learning from others. Participants were thus found to show cultural awareness and cultural sensitivity on a precultural competence level in this study. As a way forward further studies can address how cultural competence is enacted in daily practice of occupational therapists; there may also be the need for adaptations to existing OT curricula on cultural competence in more formal ways to ensure a more comprehensive understanding of the concept by exploring terms such as cultural awareness, cultural sensitivity, and cultural knowledge. Finally, we encourage the occupational therapy community to engage in critical debate on whether a state of cultural competence may ever be possible within occupational therapy practice and how this may contribute to the professions identity.

## 7. Limitations of the Study 

The study was limited to one cohort of students in a particular context and the authors did not assess or observe actual behaviours of the students within the framework of cultural competence. Thus, participants may have recalled and expressed their experiences differently to their actual behaviour or attitudes.
